# Activation of the stress response among the cardiac surgical residents: comparison of teaching procedures and other (daily) medical activities

**DOI:** 10.1186/s13019-022-01873-z

**Published:** 2022-05-11

**Authors:** George Awad, Robert Pohl, Sabine Darius, Beatrice Thielmann, Sam Varghese, Max Wacker, Hendrik Schmidt, Jens Wippermann, Maximilian Scherner, Irina Böckelmann

**Affiliations:** 1 Department of Cardiothoracic Surgery, Otto-von-Guericke University Magdeburg, Leipziger Strasse 44, 39120 Magdeburg, Germany; 2 Department of Occupational Medicine, Otto-von-Guericke University Magdeburg, Magdeburg, Germany; 3 Clinic for Cardiology and Diabetology, Magdeburg Clinic, Magdeburg, Germany; 4 University Clinic for Internal Medicine III, Martin Luther University Halle-Wittenberg, Halle, Germany

**Keywords:** Cardiac surgery, Education, Workload, Stress, Heart rate variability

## Abstract

**Background:**

The aim of this Pilot study was to investigate the cardiac surgical residents’ workload during different surgical teaching interventions and to compare their stress levels with other working time spent in the intensive care unit or normal ward.

**Methods:**

The objective stress was assessed using two cardiac surgical residents’ heart rate variability (HRV) both during surgical activities (32 selected teaching operations (coronary artery bypass graft n = 26 and transcatheter aortic valve implantation n = 6), and during non-surgical periods. Heart rate, time and frequency domains as well as non-linear parameters were analyzed using the Wilcoxon test.

**Results:**

The parasympathetic activity was significantly reduced during the surgical phase, compared to the non-surgical phase: Mean RR (675.7 ms vs. 777.3 ms), RMSSD (23.1 ms vs. 34.0 ms) and pNN50 (4.7% vs. 10.6%). This indicates that the residents had a higher stress level during surgical activities in comparison to the non-surgical times.

The evaluation of the Stress Index during the operations and outside the operating room (8.07 vs. 10.6) and the parasympathetic nervous system index (− 1.75 to − 0.91) as well as the sympathetic nervous system index (1.84 vs. 0.65) confirm the higher stress level during surgery. This can be seen too used the FFT Analysis with higher intraoperative LF/HF ratio (6.7 vs. 3.8).

**Conclusion:**

HRV proved to be a good, objective method of identifying stress among physicians both in and outside the operating room. Our results show that residents are exposed to high psychological workloads during surgical activities, especially as the operating surgeon.

## Introduction

Physicians in operating theaters are exposed to various risks and occupational hazards such as unfavorable body posture [1], anesthetic gases and electrosurgical smoke plumes [2], radiation, disturbing noise and infections [3]. In addition, surgeons complain about high psychological stress levels during working hours [4].

Today, preventive medicine and occupational health research are concerned with searching for stress parameters, which can detect psychological disorders and mental illness at an early stage. However, higher psychological strain and workloads have also been reported among surgeons. In comparison to other medical disciplines, they work longer and have a lower quality of life [5].

Another study, which analyzed psychosocial stress at work in various surgical departments in Germany, revealed that this group of physicians suffers from more severe stress at work than other occupational groups. Furthermore, one quarter of hospital doctors who perform surgery experience an occupational gratification crisis [6]. This study was carried out using the Job-Demand-Control Model proposed by Karasekand Siegrist’s model of professional gratification crises [7].

In fact, many versions and concepts (including Rohmert and Rutenfranz’s stress–strain model [8]) have been established by research to identify stress in different occupational groups and to clarify the relationship between work and illness or health [9]. While evaluating the workload and strain were challenging, the blood pressure, heart rate (HR) and heart rate variability (HRV) are considered to be objective determinants of stress [10, 11]. Also, current neurobiological evidence suggests that HRV is influenced by stress and supports its use for objective assessment of psychological health and stress [12].

A psychophysiological multi-level approach is recommended to allow a better grasp of the *mental stress—individual—mental strain* reaction chain’s complexity. This enables the mental stress and occurred strain [13, 14] to be evaluated, as well as the collection of objective performance characteristics and subjective and objective stress data.

Heart rate variation is considered to be the result of the different regulatory mechanisms which control the homeostasis of the cardiovascular system, being activated. HRV is one of the most informative stress parameter for estimating or assessing the state of a patient´s autonomic function. For example, a lower HRV is associated with a weak cardiovascular system adaptability and is therefore not ideal for the organism.

Recently, 17 studies which observed the use of HRV to measure mental stress among surgeons were collected and analyzed [15]. This systematic review showed that HRV is a good method for detecting and determining stressors during operations. In addition, the existing studies of the determination and assessment of workloads in surgical disciplines, show that the collection of stress parameters (mainly parameters from HRV analyses) is essential for the evaluation of surgeons’ intraoperative conditions [10, 16, 17].

In two major reviews of various studies relating to surgeons’ mental stress between 1980 and 2015, Thielmann and Boeckelmann [10] and Arora et al. [18] confirmed that the HR increases in stressed surgeons and the HRV decreases.

According to the current state of literature, more research is needed in this area. Therefore, we aimed to objectively investigate the workloads of the cardiothoracic surgical residents during different phases of their working day, by using HR and HRV parameters. Nonlinear analysis and HRV parameters from the time and frequency domain were used for this purpose.

In addition, we also sought to compare the young surgeons’ workload during training in coronary artery bypass graft (CABG) procedures and while learning transcatheter aortic valve implantation (TAVI) with their normal daily medical activities (intensive care unit or normal ward) in terms of a Pilot study.

## Materials and methods

### Study design, participants and operational procedure

The objective workload and stress experienced by surgical residents during various times at their workplace were observed as part of a cooperation between the occupational medicine and cardiothoracic departments of the Otto-von-Guericke-University Magdeburg.

Two residents from the department of cardiothoracic surgery volunteered as test subjects. They had been in the cardiac surgery training program for 7 years and were between 30 and 35 years old. They were healthy with no previous conditions or medication.

The two test subjects signed declarations of consent and completed questionnaires for the collection of socio-demographic data. A portable Holter-2-channel electrocardiogram (ECG) (model MT-101, Schiller AG, Switzerland) was for 24-h applied during 32 selected surgical teaching interventions (on-pump coronary artery bypass graft (CABG = 26); transaortic valve implantation (TAVI = 6)) and during their work in the intensive care unit or on a normal ward. The heart rates were collected over an 8-month period and the HRV was analyzed. The data for the two work phases, i.e. the intraoperative and the non-surgical phases, were compared.

A distinction was made in the intraoperative phase, between two different procedures in which the test residents were working as surgeons or as assistants. During the CABG operations, the two residents performed as surgeons, supervised by a senior surgeon. In the other cases, the residents assisted in the TAVI procedure, while an attending performed the intervention. Data was acquired during the CABG and TAVI procedures at predefined time points.

The non-surgical time points were recorded outside the operating theatre on surgery days or on other working days, when the test residents were working in the intensive care unit or on the surgical medical floor. This data was documented using a protocol performed by the resident himself. The duration of the non-surgical phase was similar to the intraoperative, as required by the HRV analysis quality criteria.

### HRV analysis

The database consisted of 32 ECG recordings (n = 19 from resident 1 and n = 13 from resident 2). All the ECGs were recorded using a 2-channel ECG device, which could identify each QRS complex and record all the RR intervals with a sampling rate of 1,000 Hz. The AWMF-s2k guideline for quality criteria was considered [11, 19]. The collected data were transferred and analyzed using the Medilog DARWIN 2 Enterprise software (version 2.9.2; Fa. SCHILLER, Switzerland), whereby artefacts were manually verified and excluded. The NN-intervals were then analyzed (NN = RR without any artefacts).

All recordings of successive NN-intervals were exported into a text file (txt format) and then analyzed using the Kubios HRV Premium program (Kubios, Kuopio, Finnland) [20, 21]. In accordance with the recommendations by the Task Force of the European Society of Cardiology and the North American Society of Pacing and Electrophysiology, the time and frequency domain analysis with non-linear methods were selected as the informative stress und load indicators in this study.

An artefact correction was performed with the settings 0.3 and custom (or smoothing, i.e. all NN-intervals that deviate from the one before by more than 30%, are eliminated as artifacts) without changing the trend components. A Fast Fourier Transform (FFT) was selected for the frequency analysis. A window width setting was selected of the time period of the analysis range and a 50% window overlap.

Frequency bandwidths (Table 1) were used in accordance with the international and national recommendations and guidelines [11, 22, 23].

**Table 1:** Explanation of the examined spectral bands

Spectral band	Definition and description	Physiological interpretation
VLF	Very low frequency or thermoregulation band: Power spectral density (PSD) in the frequency range up to 0.04 Hz (≤ 0.04 Hz), period duration from 25 s to 5:30 min, here the sympathetic nervous system is involved	Thermoregulation related fluctuations, hormonal rhythms
LF	Low Frequency Power or Baroreflector band: Power spectral density (PSD) in the frequency range 0.04–0.15 Hz, period duration from 7 to 25 s; here both the sympathetic and the parasympathetic nervous system are involved	Blood pressure related fluctuations
HF	High Frequency Power or Respiratory sinus arrhythmia, respiratory band: Power spectral density (PSD) in the frequency range 0.15–0.40 Hz, period duration from 2.5 bis 7 s; shows only the parasympathetic function	Breathing related fluctuations

**Fig. 1:**
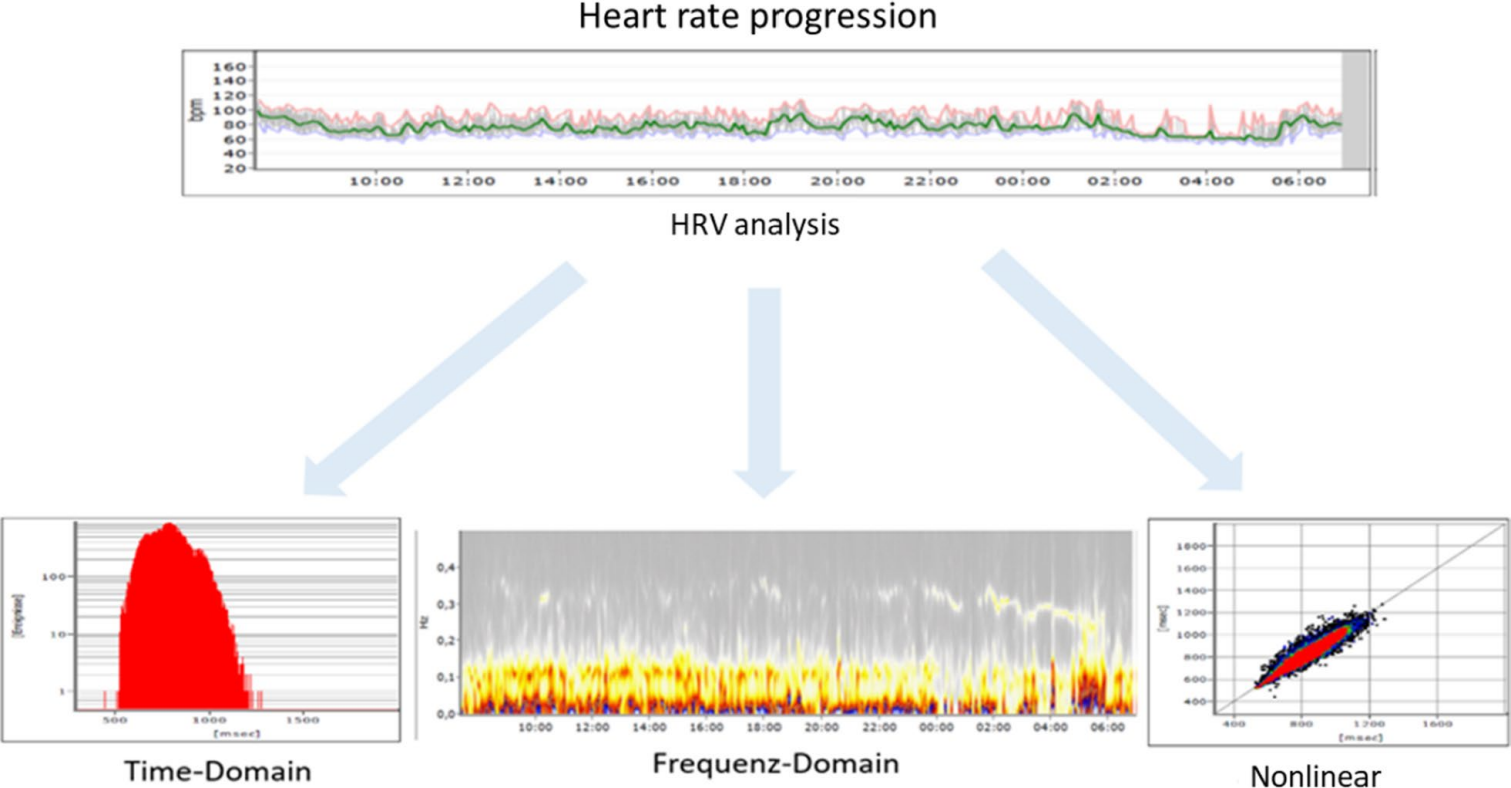
Overview of the options for HRV analyses

The HRV analysis was performed used three different methods: (Fig. 1).

#### Time-domain measurements

The NN intervals were evaluated with respect to their variance. The monitoring periods ranged from 1 min to 24 h. A few time-related parameters were attributed to both sympathetic and parasympathetic activity, and another does not have a clear classification (Table 2) [22].

**Table 2:** Explanation of HRV parameters by Time–Frequency-Domain and nonlinear Parameters (according to [19, 25])

HRV-parameter	Description	Activity as part of the autonomic nervous system	Reaction with increased stress
*Time-domain*			
Mean HR (1/min)	The mean heart rate	Sympathetic and parasympathetic nervous system (SNS and PNS)	↑
STD HR (1/min)	Standard deviation of instantaneous heart rate values		
Mean RR or NN (ms)	The mean of RR intervals	Unclear classification	↓
SDNN (ms)	Standard deviation of RR intervals	SNS and PNS	↓
RMSSD (ms)	Square root of the mean squared differences between successive RR intervals	PNS	↓
NN50	Number of successive RR interval pairs that differ more than 50 ms	PNS	↓
pNN50 (%)	NN50 divided by the total number of RR intervals	PNS	↓
RR_Tri	The integral of the RR interval histogram divided by the height of the histogram	Unclear classification	
TINN (ms)	Baseline with of the RR interval histogram	Unclear classification	↓
*Frequency-domain (FFT)*			
FFT VLF-peak (Hz)	VLF band peak frequencies	Unclear classification	No information
FFT LF-peak (Hz)	LF band peak frequencies	Unclear classification	No information
FFT HF-peak (Hz)	HF band peak frequencies	Unclear classification	No information
FFT_VLF_po (ms^2^)	Absolute powers of VLF bands	SNS	↑
FFT_LF_po (ms^2^)	Absolute powers of LF bands	SNS and PNS	↑
FFT_HF_po (ms^2^)	Absolute powers of HF bands	PNS	↓
FFT TP (ms^2^)	Total Power: Overall Power spectral density: from 0.00001 to 0.4 Hz	Unclear classification	↓
FFT_LF_po% (%)	Relative power of LF bands	SNS and PNS	↑
FFT_HF_po% (%)	Relative power of HF bands	PNS	↓
FFT LF nu	Low frequency normalized unit: LF/(Total Power-VLF) × 100	SNS and PNS	↑
FFT HF nu	High frequency normalized unit: HF/(Total Power − VLF) × 100	PNS	↓
FFT LF/HF	Ratio between LF and HF band powers	SNS and PNS	↑
*Nonlinear*			
SD1 (ms)	The standard deviation of the Poincaré plot perpendicular to (SD1) and along (SD2) the line-of-identity	PNS	↓
SD2 (ms)	The standard deviation of the Poincaré plot perpendicular to (SD1) and along (SD2) the line-of-identity	SNS and PNS	
DFA1	Detrended fluctuation analysis: Short term fluctuation slope	Unclear classification	
DFA2	Detrended fluctuation analysis: Long term fluctuation slope	Unclear classification	

#### Frequency-domain measurements

The most common methods of analyzing frequency-related parameters are spectral analysis using nonparametric Fast Fourier Transform (FFT) or Parametric Auto-Regression (AR) [11, 22]. The FFT or AR separate the HRV into ULF, VLF, LF and HF rhythms, which operate in several different frequency ranges (Table 2) [24].

#### Nonlinear measurements

The ECGs yield a series of successive NN-intervals. The time intervals between the heartbeats are variable and the relationship between these variables cannot be mapped as a straight line. Nonlinear methods, such as a Poincaré plot (Lorenz-Plot) or detrended fluctuation analysis (DFA), were used to examine the variability of the successive NN-intervals [22]. Both methods are explained and well described [24].

Table 2 gives an overview of the individual HRV parameters differentiated by time, frequency, and nonlinear ranges.

### Parameters of the sympathetic and parasympathetic nervous system and the stress index

The stress index is based on the values in Baevsky’s Stress Index and shows how long the test subjects were in stress zones during a recording. To gain an overview of the subjects’ HRV parameter levels, six HRV parameters are presented, divided into parasympathetic nervous system (PNS) tone and sympathetic nervous system (SNS) tone [25]. A description of these parameters is given in Table 3.

**Table 3:** Explanation of HRV parameters according to the Kubios Manual [25]

HRV-parameter	Description	Activity as part of the autonomic nervous system	Reaction with increased stress
*Overview stress parameter*			
Stress index	The square root (to make the index normally distributed) of the Baevsky’s stress index	SNS	↑
PNS index	Parasympathetic nervous system activity compared to normal resting values (PNS tone described by Mean RR, RMSSD and SD1(%)	PNS	↓
SNS index	Sympathetic nervous system activity compared to normal resting values (SNS tone described by Mean HR, Stress Index and SD2(%))	SNS	↑

### Statistical analyses

The statistical analysis was performed using the IBM SPSS software package 25 (IBM Corp., Somers, NY). The Kolmogorov–Smirnov test was used to check the interval-scaled data for normal distribution. If the data were not normally distributed, the paired-sample Wilcoxon test was used for nonparametric tests. A value < 0.05 was considered to indicate statistical significance.

## Results

Table 4 presents an overview of the time-related HRV parameters and the respective significance levels (p). The mean values (Mean) and the standard deviations (SD) of the HRV parameters from the intraoperative periods are compared with those from the non-surgical periods.

**Table 4:** Comparison of time-related HRV parameters between intraoperative times (for CABG and TAVI) and non-surgical times (N = 32)

Variable	Intraoperative times	Non-surgical times	p value
	Mean ± SD	Mean ± SD	
Mean RR (ms)	675.7 ± 56.57	777.3 ± 102.67	< 0.001
SDNN (ms)	40.6 ± 12.34	43.6 ± 7.22	0.112
Mean HR (1/min)	89.4 ± 7.71	78.5 ± 10.07	< 0.001
STD_HR (1/min)	4.34 ± 1.73	4.6 ± 1.43	0.135
RMSSD (ms)	23.1 ± 8.16	34.0 ± 6.25	< 0.001
NN50	816.7 ± 743.13	1786.2 ± 1059.80	< 0.001
pNN50 (%)	4.7 ± 4.58	10.6 ± 4.56	< 0.001
RR_tri	10.6 ± 4.45	10.6 ± 1.82	0.350
TINN (ms)	296.94 ± 63.66	3406 ± 47.23	0.006

The low values for mean RR (675.7 ms vs. 777.3 ms; p < 0.001), RMSSD (23.1 ms vs. 34.0 ms; p < 0.001), NN50 (816.7 vs. 1786.2; p < 0.001), pNN50 (4.7% vs. 10.6%; p < 0.001) and TINN (296.94 ms vs. 340.6 ms; p < 0.001) revealed that the residents had significantly higher levels of stress during surgical activities, compared to non-surgical activities. This can also be seen in the higher mean HR value of 89.4 vs. 78.5 min^−1^ (p < 0.001). For the parameters SDNN, STD_HR and RR_tri, no statistically significant differences were found between the surgical- and non-surgical times. However, the average RMSSD values of 23.1 ms during surgery are still considered to be low. The frequency-related HRV parameters from the FFT are shown in Table 5.

**Table 5:** Comparison of frequency-related HRV parameters from the FFT analysis between intraoperative times (for CABG and TAVI) and non-surgical times (N = 32)

Variable	Intraoperative times	Non-surgical times	p value
	Mean ± SD	Mean ± SD	
FFT_VLF_pe (Hz)	0.0289 ± 0.01430	0.03419 ± 0.0041	0.562
FFT_LF_pe (Hz)	0.0899 ± 0.0152	0.0870 ± 0.0214	0.466
FFT_HF_pe (Hz)	0.160 ± 0.0092	0.178 ± 0.0468	0.513
FFT_VLF_po (ms^2^)	450.2 ± 817.59	152.5 ± 61.84	0.477
FFT_LF_po (ms^2^)	1139.2 ± 585.32	1420.64 ± 582.01	0.030
FFT_HF_po (ms^2^)	200.9 ± 139.69	411.3 ± 195.87	< 0.001
FFT_Total_po (ms^2^)	1791.4 ± 942.30	1985.6 ± 764.76	0.191
FFT_VLF_po% (%)	18.3 ± 24.74	8.4 ± 3.41	0.926
FFT_LF_po% (%)	69.9 ± 20.74	71.2 ± 5.73	0.184
FFT_HF_po% (%)	11.7 ± 5.01	20.4 ± 5.11	< 0.001
FFT_LF_po_nu (n.u.)	86.06 ± 3.55	77.71 ± 5.53	< 0.001
FFT_HF_po_nu (n.u.)	13.88 ± 3.55	22.23 ± 5.51	< 0.001
FFT_LF/HF	6.7 ± 2.06	3.8 ± 1.27	< 0.001

The HF band analyses, which represent the parasympathetic activity, showed that the mean of the power spectrum with 200.9 ms^2^ and the relative part of the total power of 11.7%, was lower during the surgical periods than during non-surgical (411.3 ms^2^ and 20.4%; p < 0.001). The low level intraoperative indicates lower vagal activity during this phase. The higher value for the LF po nu (86.06 to 77.71) and the lower HF po nu (13.88 to 22.23) during the surgical times, are also attributed to the increased stress during operations. The LF/HF ratio is higher, on average 6.7, for the operating times, compared to 3.8 for non-surgical times, which also proves that there is a higher level of stress and thus a higher stress and load situation, in the operating theater.

Table 6 illustrates the nonlinear parameters of this study.

**Table 6:** Comparison of the nonlinear HRV parameters between intraoperative times (for CABG and TAVI) and non-surgical times (N = 32)

Variable	Intraoperative times	Non-surgical times	p value
	Mean ± SD	Mean ± SD	
SD1 (ms)	16.3 ± 5.77	24.1 ± 4.42	< 0.001
SD2 (ms)	54.8 ± 17.32	56.7 ± 9.70	0.231
DFA1	1.51 ± 0.071	1.33 ± 0.083	< 0.001
DFA2	0.53 ± 0.229	0.42 ± 0.044	0.018

SD1 is an indicator of short-term variability and SD2 is an indicator of long-term variability (Table 2). Regarding short-term variability, the SD 1 parameters are significantly different for surgical and non- surgical time. The RR intervals’ low dispersion (16.3 ms vs. 24.1 ms) during the operations, reveals a limited regulation in the residents intraoperatively.

Both the DFA values are above the normal range of 1.0 and significantly different in the two phases investigated (p < 0.001 and 0.018). With 1.51, the DFA1 in the surgical phases shows a higher correlation to the short-term fluctuations of the NN intervals compared to the non-surgical phases, with 1.33. The lower DFA 2 in the non-surgical phase, indicates a poor distribution of the NN-intervals.

Table 7 shows the parameters of the stress index, the PNS- and SNS index.

**Table 7:** Comparison of stress HRV parameters (stress index, PNS and SNS) between intraoperative times (for CABG and TAVI) and non-surgical times (N = 32)

Variable	Intraoperative times	Non-surgical times	p value
	Mean ± SD	Mean ± SD	
PNS index	− 1.75 ± 0.40	− 0.91 ± 0.53	< 0.001
SNS index	1.84 ± 0.72	0.65 ± 0.77	< 0.001
Stress index	10.62 ± 2.14	8.07 ± 0.92	< 0.001

All these three parameters differ significantly in the two phases (p < 0.001). The activity of the parasympathetic component, which is indicated by the PNS index, is lower during the operations than in the non-surgical phase (− 1.75 to − 0.91; p < 0.001). The activity of the sympathetic part, which reflected by the SNS index, is higher intraoperatively, with 1.84, compared to 0.65 outside the operating room (p < 0.001).

The stress index is lower in the non-surgical phase, which reflects a lower stress level during this time. However, the stress index with average values of 8.07 (non-surgical phase) and 10.6 (surgical phase), are both within the normal range (7.1–12.2; p < 0.001).

## Discussion

The results of our single-center, observational Pilot study shows that the cardiac surgical residents have significantly higher sympathetic activity as operators during surgical procedures than outside the operating room. This was proven by several stress and load parameters, especially HRV, which was recommended by the current scientific studies and reviews in this field [10, 16, 26, 27].

Many of the residents’ psychological and psycho-emotional burdens are not restricted to the operating room, but also occur in the intensive care unit or surgery. Other potential burdens on surgeons may include night shifts, responsibility towards patients or superiors [28], high bureaucracy [29], and very long working hours [30]. Residents are therefore exposed to a large health risk. In the literature, a study by Raspe et al. reported on the increased use of illicit and prescription drugs among young physicians due to high workload [29], and in a study by Franke et al., specifically among surgeons. This can be understood as a consequence of the high workload [31].

Based on our observations, we could prove that the residents’ heart rates were significantly higher during operations than during the non-surgical phases. This has also been observed in other studies associated with HR analyses during surgery [32–34].

Recording the HR as the unique parameter for the description of a stress situation is now considered a very limited method for making adequate statements about psychological stress [35]. However, the HRV analyses are appropriate for determining stress in the psychophysiological studies, as they allow a more specific statement to be made about the stress level, as well as the cardiovascular system’s competence and regulatory ability [11, 22].

Different studies have mainly used spectral analyses with nonparametric FFT or parametric AR, as well as measurements in the time domain, to assess psychological stress among surgeons. However, so far, nonlinear analyses have rarely been used in research studies of surgeons [15].

The intraoperative stress parameters collected in our study illustrate the residents’ high stress levels during surgical training procedures. The parasympathetic parameters are significantly lower during surgical activities than during other medical activities. This also applies to the sympathetic parameters, which are higher during an operation than during the non-surgical phases. For example, for the physicians, tasks on a normal ward or in ICU or talking with the patient's relatives, are not as stressful as the surgical interventions.

This is illustrated by the low PNS activity, based on the reaction of the Mean RR, RMSSD and SD1(%) parameters and the higher tone of the SNS (based on the changes in the Mean HR, Stress Index and SD2 (%) parameters (Tables 4, 6).

Considering the RMSSD zones proposed by M. P. Tarvainen et al. [25], the residents’ RMSSD values can be classified as being in the *lower zone* (12–27 ms) during surgery and in the *normal zone* (27–72) outside the operating room. Within the stress zone, the residents' values were average (7.1–12.2) [25].

Low parasympathetic activity is associated with an increased level of stress [22]. Prichard et al. [36] showed the same results when they compared two groups of endocrine surgical consultants and fellows, who performed 50 thyroid lobectomies as primary operators and 50 as assistants in a cross-over study design. The HRV analysis revealed that members of the fellows’ group had higher stress levels during surgery than when assisting. Although there was a lack of statistical significance, this was shown by lower RMSSD and pNN50 parameters and a higher SDNN, as well as a higher LF/HF ratio.

In Rieger et al. [16] the surgeons were allocated to a stressed and a non-stressed group based on their perceived stress (State Trait Anxiety Inventory). The interoperative stress was measured using HRV and the changes in autonomic nervous system activity were quantified by frequency and time domain analysis of RR interval variability. The stressed surgeons showed low intraoperative values for RMSSD, SDNN and pNN50, compared to the non-stressed group [16]. Using the time-related HRV parameters, Stress-, parasympathetic nervous System—and sympathetic nervous System Index illustrated that the residents showed higher stress levels than their supervising seniors during CABG teaching procedures [37].

The HF band is associated with parasympathetic (vagal) activity [22, 23]. This explains the significantly lower power and relative share of the HF band in our study, as well the residents’ higher power and relative share of the LF band during surgical procedures.

The LF/HF ratio has been used in other studies to make statements about the interaction between the parasympathetic nervous system (HF) and the sympathetic nervous system (LF). On the assumption that LF power increases with increasing sympathetic activity and HF power increases with increasing vagal activity, Song et al. (2009) investigated psychological stress during cardiosurgical bypass operations by using the ratio of LF to HF (LF/HF ratio) [38]. The residents (less experienced surgeons) exhibited higher LF/HF ratio, mainly in the ischemia phases (cross-clamp time). The residents in our study had an LF/HF ratio of 6.7 in the FFT, which is slightly below the values reported in the Song et al. 2009 study but still far higher that the LF/HF ratio of 1.5–2.0 which is defined as normal [23].

In the study conducted by Demirtas et al. [35], the HRV parameters of the surgeon and his first assistant during rhinoplasty procedures were compared with rest periods [35]. The results obtained were similar to those obtained in our study. For both surgeons and assistants, the sympathetic activity is higher in the OR than during the non-surgical phases. Nevertheless, it is possible to observe a higher stress level in the surgeons than in their assisting surgeons.

Yamanouchi et al. [39] used frequency domain methods with surgeons before, during and after surgical activities, to make statements about parasympathetic and/or sympathetic activity and therefore about the respective psychological stress level. Although the surgeons’ experience was not taken into account, it was shown that there are significantly lower HF components and higher LF components in the HRV analyses during surgery. The resulting higher LF/HF ratio reflects the higher psychological stress during the operations [39]. This project also demonstrated that an increase in sympathetic activity was mainly observed during critical phases of surgery.

The small sample of two residents in our study has limited significance. Therefore, the results should not be generalized and it would be desirable to sensitize physicians and especially surgical residents to more studies of this kind. Moreover, the surgical procedures used in our study were very different. While the residents mainly assisted in the TAVI procedures, in the CABGs, the young surgeons were operating themselves, under the supervision of a consultant. In addition, there are various factors that can have different effects on the residents’ stress levels in the OR, such as unexpected bleeding or each patient’s different risk factors. Therefore, this study can be considered as a pilot study that addresses important questions concerning the health consequences of the stressful situations faced by surgeons and might lead the surgical community to pay attention to these aspects of surgical training programs. In this context, we like to pint out that the teaching situation is special in cardiac surgery due to the particular process of the procedures: At first, most of the possible complications during the procedures are potentially acute life threatening (e.g. aortic dissection during cannulation; myocardial infarction due to insufficient anastomosis etc.) and secondly, the time aspect is of major importance as the cross clamp time is directly related to patients outcome. Having these facts in mind, teaching and learning in cardiac surgery is special and requires a long learning period. This does not mean, that the demands in other surgical specialities are less challenging and it would be interesting and useful to investigate stress response in every surgical (and medical) field.

As mentioned above, we cannot generalize the results based on the data of only two residents. The findings of our study should be investigated and verified in future studies with a larger sample size. As cardiac surgery training programs often show a limited number of trainees, a multicenter setting might be most useful to further investigate this topic. Nevertheless, the purpose of this publication is also to raise awareness of this topic among cardiac surgeons and the surgical community in general.

One of the strengths of this study is the HRV recording method with using 3-channel ECG devices with automatic detection of the R peaks over 24 h and a set sampling frequency of 1.000 Hz. This increased accuracy in detecting the RR intervals and reduced problems with artifacts detection.

Nevertheless, the results are consistent with those of other research contributions on intraoperative stress analysis using HRV in surgeons, and confirm the demonstrably high risk of burnout in the medical professions [40–42]. In addition, the various effects of long-term work-related stress on health, such as cardiovascular diseases [43], diabetes mellitus type 2 [44] and depression [45], underline the need for action/treatment from an occupational health perspective.

The fact that young surgeons demonstrated high levels of stress in the operating theatre can be considered as an initial potential starting point for early sensitization concerning mental load of both medical students and inexperienced surgical residents. In this context, it is important to act on an institutional and individual level to generate awareness, which can help to counteract the high level of stress they will experience later in their professional lives.

## Data Availability

Basic data are saved in the Department of Occupational Medicine, Otto-von-Guericke University Magdeburg and can be made available on request.
